# Supplementary material for: A possible route towards dissipation-protected qubits using a multidimensional dark space and its symmetries
Source: Nat Commun. 2020 Nov 19;11:5899. doi: 10.1038/s41467-020-19646-4 (PMC7677546; doi:10.1038/s41467-020-19646-4)
Supplement: Supplementary file 1 — Supplementary Information [file 41467_2020_19646_MOESM1_ESM.pdf]

Supplementary Information for:
A possible route towards dissipation-protected qubits using a multidimensional dark space and its symmetries

Santos, et al.

In this Supplementary Information we complement and expand some of the discussion in the main text. We analyse of the Lindbladian gap of the dissipative model Supplementary Note 1 . The role of perturbations is discussed in Supplementary Note 2 . In Supplementary Note 3 we show details of the adiabatic evolution analysis. Finally we include a longer discussion about the experimental realization of the Lindblad operators.

The dissipative evolution $\dot{\rho} = \mathcal{L}(\rho)$ with $\hat{H} = 0$ studied in the main text is defined by the quantum jump operators $\tilde{\ell}_i = R_i^\dagger \mathcal{Q}_i$ for $i = 1, ..., L$ with $R_i^\dagger = c_i^\dagger c_{i+1}^\dagger + t(\ell_{0,i}^\dagger + \ell_{0,i+1}^\dagger)$ and

$$\begin{aligned} \mathcal{Q}_i &= \ell_{1,i} + A(\ell_{0,i-1} + \ell_{0,i+1}) + B(\ell_{1,i+1} + \ell_{1,i-1}) \\ \ell_{0,i} &= c_i c_{i+2}, \qquad\qquad \ell_{1,i} = c_i c_{i+1} + \beta c_{i-1} c_{i+2}. \end{aligned} \tag{1}$$

Supplementary Note 1 . LINDBLADIAN GAP IN FINITE SYSTEM SIZES

We show here our analysis for the dissipative gap of the Lindbladian for finite system sizes. We obtain the gap in two different forms: (i) directly by exact diagonalization of the Lindbladian superoperator, or (ii) indirectly by the asymptotic decay rate (ADR) of the quantum state dynamics. While exact diagonalization allows us to study the Lindbladian gap for sizes up to $L \sim 12$, the asymptotic decay rate analysis allows the study of larger $L \sim 15$ system sizes.

The exact diagonalization analysis is performed by first describing the Lindbladian superoperator as a linear operator in the extended Hilbert space,

$$\mathcal{L} \to |\mathcal{L}\rangle = -i(\mathbb{I} \otimes \hat{H} - \hat{H}^T \otimes \mathbb{I}) + \sum_i \hat{\ell}_i^* \otimes \hat{\ell}_i - \frac{1}{2}\mathbb{I} \otimes \hat{\ell}_i^\dagger \hat{\ell}_i - \frac{1}{2}(\hat{\ell}_i^\dagger \hat{\ell}_i)^T \otimes \mathbb{I} \tag{2}$$

and then diagonalizing the vectorized Lindbladian $|\mathcal{L}\rangle$. The eigenvalues λ_j of the Lindbladian have non-positive real values, with the DDS described by those with zero eigenvalue. The gap of the Lindbladian corresponds in this way to the eigenvalue with largest nonzero real part. Ordering the eigenvalues according to their real part $\Re(\lambda_j) \geq \Re(\lambda_{j+1})$, for $j = 1$ to the extended Hilbert space dimension, the dissipative model of this work have in this way $\lambda_j = 0$ for $j = 1, ..., 9$ and the gap is described by the eigenvalue λ_{10}.

On the other hand, one can also extract the gap by studying the dynamics of the quantum state. In the long time limit only the slower decaying modes of the Lindbladian are relevant to the dynamics and the expectation value of any observable $O(t) = Tr(\hat{O}\hat{\rho}(t))$ is approximated by

$$O(t) - O(t \to \infty) \approx e^{\lambda_{\rm ADR} t} \tag{3}$$

where $\lambda_{\rm ADR} < 0$ corresponds to the slower decay mode (or asymptotic decay mode) for the observable $\hat{O}$.

In Supplementary Figure 1 we show our results for the Lindbladian gap and ADR analysis of the DDS(t) dynamics. We observe that the gap from exact diagonalization matches the one obtained from ADR. Furthermore we see that the dissipative gap increases going from 9 to 12 sites, and decreases again for 15 sites (to slightly the same value as 9 sites), which is the limit size that we can achieve. Although instructive, these numerical results for small systems do not allow us to unequivocally determine the nature of the dissipative gap in the thermodynamic limit. It is suggestive, however, for the presence of either (i) a gapped Lindbladian in the thermodynamics limit, or (ii) a gapless Lindbladian with a slow decaying of the gap with system size, excluding e.g. the possibility of an exponential closure of the gap with system size which would be detrimental for the preparation and manipulation of DDS in quantum information tasks.

In the main text, we showed that the existence of the symmetry operators U and T ensures that all of the eigenvalues of the Lindbladian are at least m-fold degenerate. It is good to stress that the eigenmatrices of the Lindbladian do not translate directly into density matrices. In particular, this means that the symmetry operators U and T do not generate directly a symmetry on the density matrices. The Lindbladian is a linear operator acting on the extended Hilbert space $V = \mathcal{H} \otimes \mathcal{H}$, (Supplementary Eq. 2) which for concreteness we can take to be spanned by the vector states $|i\rangle|j\rangle$, each defined within a copy of $\mathcal{H}$. The vectorized density matrices form a subspace $S \subset V$ which is not a vector space, as the sum of two elements of S does not generically belong to S (e.g. given $\rho_{1,2}$ two positive definite operators with unit trace, the linear combination $\alpha\rho_1 + \beta\rho_2$ with α, β complex numbers is not necessarily positive definite).

Supplementary Note 2 . LINDBLADIAN PERTURBATIONS

In this section we study the effects of Lindbladian imperfections on the DDS. We consider the following different imperfections:

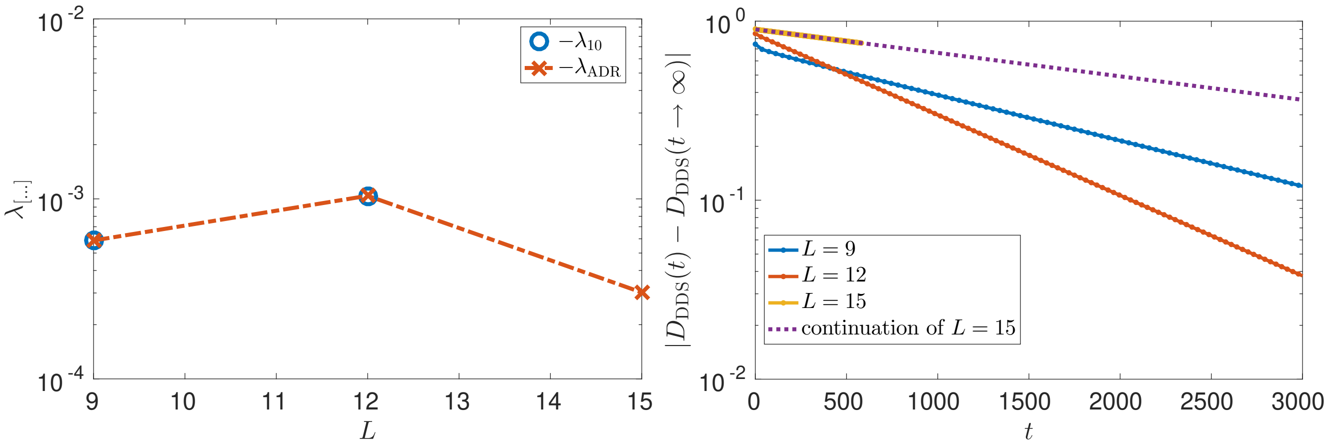

FIG. Supplementary Figure 1. **Lindbladian gap for finite system sizes.** In the **left-panel** we show our results for the Lindbladian gap (gap $= \lambda_{10}$) obtained from exact diagonalization of the vectorized Lindbladian (Supplementary Eq.(2)), and the asymptotic decay rate extracted from the dynamics of $D_{\rm DDS}(t)$, for varying system sizes. In the **right-panel** we show the dynamics of $D_{\rm DDS}(t)$ for a quench dynamics starting from an initial CDW state. We show the dynamics in a log-linear scale highlighting the exponential asymptotic behavior towards the steady state value $D_{\rm DDS}(t \to \infty) = 1$. The dashed line corresponds to the expected long time dynamics for the $L = 15$ case, according to its ADR coefficient. The Lindbladian parameters in all figures are $A = B = t = \beta = 1$.

- Additional set of dephasing dissipative channels:

$$\hat{\ell}_{j,\rm dephasing} = \sqrt{\epsilon_{\rm deph}}\left(2\hat{c}_j^\dagger \hat{c}_j - 1\right), \qquad j = 1, ..., L \tag{4}$$

describing fluctuations of on-site energies, which tend to suppress coherences between classical particle number basis of the fermionic system.

- Imperfections in the current Lindblad operators of the form of extra hopping terms:

$$\tilde{\ell}_j \to \hat{\ell}_{j,\epsilon} = \tilde{\ell}_j + \sqrt{\epsilon_\ell}(c_j^\dagger c_{j+1} + \text{h.c.}), \qquad j = 1, ..., L \tag{5}$$

- Coherent Hamiltonian competing with the dissipative dynamics:

$$\hat{H} = -\epsilon_H \sum_j (c_j^\dagger c_{j+1} + \text{h.c.}), \tag{6}$$

describing the case in which despite the dissipation being the leading term, there are still some non-negligible coherent local dynamics within the fermionic system. We consider the simplest form of hopping terms, but small local coherent perturbations lead to the same conclusions.

- Additional set of decay dissipative channels:

$$\hat{\ell}_{j,\rm decay} = \sqrt{\epsilon_{\rm decay}}\hat{c}_j, \qquad j = 1, ..., L \tag{7}$$

corresponding to losses of particles in the optical lattice due to an extra channel, driving the fermionic system towards an empty vacuum state

Our results for the first three imperfections above are shown in Supplementary Figure 2. We see that perturbation theory provides a qualitative picture: in the regime of small perturbations (in units of the rate of the original Lindbladian, $\epsilon \ll 1$) while imperfections in the jump operators lead to a linear splitting of the DDS, $\lambda_2 \sim \epsilon$, a Hamiltonian perturbation leads to a quadratic dependence $\lambda_2 \sim \epsilon^2$. Thus, as long as the perturbation is small compared to the unperturbed gap there is a time window between the system entering the DDS and the system characteristics of the state being destroyed by the imperfections, which gives a possibility to effectively use these states for quantum information tasks.

The case of an additional set of decay dissipative channels follows in a similar form. In this case the steady state of the evolution is the vacuum state for $\epsilon_{\rm decay} > 0$. However, as above, if the perturbation is small there is a time window over which the effects on the DDS are negligible. One may obtain the characteristic time of the dissipative decay effects by the dynamics of the total number of particles $\hat{N}$ in the system, which in the Heisenberg picture is described by $\mathcal{L}^\dagger[\hat{N}] = -\epsilon_{\rm decay}\hat{N}$, i.e., $N(t) \sim e^{-\epsilon_{\rm decay}t}$. Thus the effects of particle losses in the system are relevant for times of the order $t \sim 1/\epsilon_{\rm decay}$, similarly to the other imperfections in the quantum jump operators considered above.

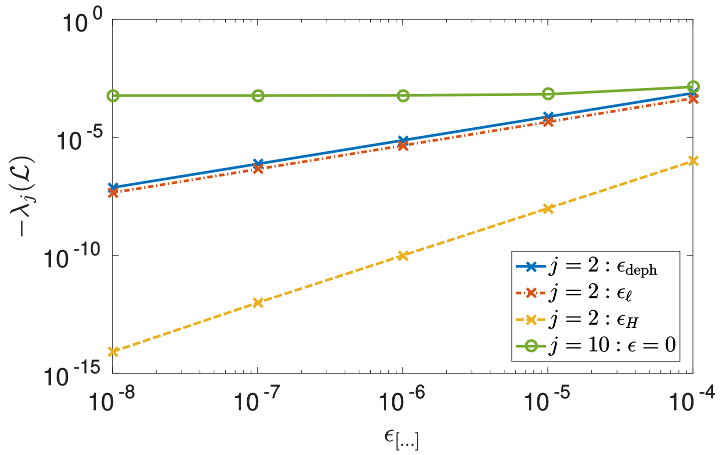

FIG. Supplementary Figure 2. **Spectral properties of the Lindbladian with imperfections.** We consider the Lindbladian of the manuscript with parameters $A=B=\beta=t=1$ for a system with $L=9$ sites and analyse the effects of the imperfections described in Supplementary Eqs.(4),(5),(6) on the spectral properties of the Lindbladian. We see that in the presence of an imperfection the DDS is not completely degenerated anymore, with a splitting of the degeneracy proportional to the imperfection strength. We obtain that $\lambda_1=0$ (by definition the Lindbladian has always a zero eigenvalue) while $\lambda_{j=2,3,\dots,9}<0$ and are approximately equal to each other. We also show in the figure for clarity the eigenvalue $\lambda_{j=10}$ for $\epsilon=0$, corresponding to the Lindbladian gap in the unperturbed case, which is also approximately equal to the cases with imperfections in the considered range of ϵ strengths.

Supplementary Note 3 . ADIABATIC EVOLUTION

In this section we expand the discussion of the adiabatic evolution in the dissipative dynamics. Using the same protocol as in the main manuscript, we evolve the system from an initial state given by a superposition of the three charge density wave configurations, precisely, $|\psi(t=0)\rangle = \left(|\Psi_{a=0}\rangle+\sqrt{2}|\Psi_{a=1}\rangle+\sqrt{3}|\Psi_{a=2}\rangle\right)/\sqrt{6}$ where $|\Psi_a\rangle$ are the Laughlin states for $\beta=0$ (i.e., product charge density wave configurations). We then evolve this state with the Lindblad operators (Supplementary Eq.(1)) using a time-dependent β parameter: $\beta(t)=\Delta\cdot t$ for $0<t\leq 1/\Delta$ and $\beta(t)=1$ for $t>1/\Delta$, where Δ is the ramp velocity.

We analyse the purity $\gamma(t)$ of the quantum state during the dynamics, as well as its overlap onto the DDS. For the later we first describe the 3×3 density matrix $\hat{\rho}_{\rm DDS}$ representing the expectation value of the quantum state in the Laughlin dark states,

$$(\hat{\rho}_{\rm DDS}(t))_{a,a'} \equiv \langle\Psi_a(t)|\hat{\rho}(t)|\Psi_{a'}(t)\rangle \tag{8}$$

for $a,a'=0,1,2$, where $|\Psi_a(t)\rangle$ are the Laughlin dark states for the Lindblad parameters at time t. We study (i) the projection of the quantum state over the DDS, given by the diagonal terms of the matrix, $D_{\rm DDS}(t)=\sum_a \rho_{\rm DDS}(t)_{a,a'}$; (ii) how the coherence of the initial state evolve under the adiabatic evolution, quantified by

$$C(t)=\sqrt{\sum_{a\neq a'}\left(\frac{\rho_{\rm DDS}(t)_{a,a'}-\rho_{\rm DDS}(0)_{a,a'}}{\rho_{\rm DDS}(0)_{a,a'}}\right)^2}, \tag{9}$$

and (iii) the distinguishability of the full matrix with the initial state, quantified by the trace norm as follows,

$$\mathcal{D}(t)=||\hat{\rho}_{\rm DDS}(t)_{a,a'}-\hat{\rho}_{\rm DDS}(0)_{a,a'}||_1 \tag{10}$$

Both coherence $C(t)$ as the distinguishability $\mathcal{D}(t)$ should be small if the evolved quantum state do not differ significantly from the initial state.

We show our results in Supplementary Figure 3. We see that for slow ramp rates the initial quantum state characteristics (purity and coherences) are preserved. We find in particular that for very slow rates $\Delta\ll 1$ the steady state properties have polynomial corrections compared to their initial conditions, i.e., $\gamma(t\to\infty)-\gamma(0)$, $C(t\to\infty)$ and $\mathcal{D}(t\to\infty)\sim\Delta^{\rm cte}$. Interestingly also to notice that the coherence is approximately constant after an initial time $t\sim O(1/\Delta)$ (i.e., $\beta(t)\sim 1$), while it is the diagonal terms of the DDS subspace ($D_{\rm DDS}(t)$) that still shows non trivial dynamics after this initial transient time.

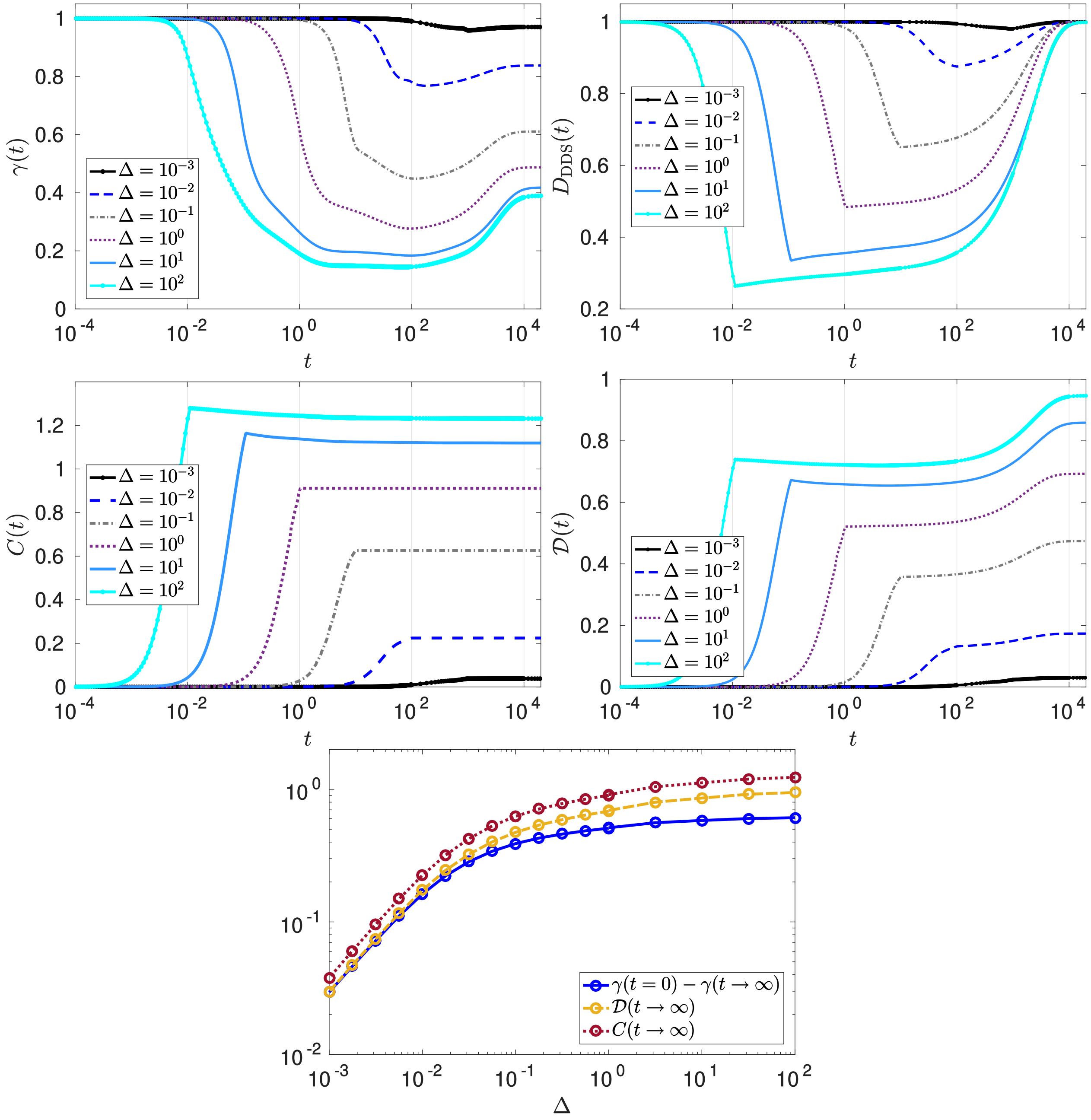

FIG. Supplementary Figure 3. **Adiabatic evolution.** We consider a system with $L = 9$ sites, $A = B = t = 1$ and varying $\beta(t)$ adiabatically according to the ramp rate Δ. The initial state of the system is given by the superposition $|\psi(t=0)\rangle = \left(|\Psi_{a=0}\rangle + \sqrt{2}|\Psi_{a=1}\rangle + \sqrt{3}|\Psi_{a=2}\rangle\right)/\sqrt{6}$. We show the dynamics of the **(top-left)** purity, **(top-right)** $D_{\mathrm{DDS}}(t)$, **(middle-left)** coherence $C(t)$ and **(middle-right)** distinguishability $\mathcal{D}(t)$. In the **bottom** panel we show their steady state values.

Supplementary Note 4 . LASER DRIVING AND COUPLING TO A BATH

We are interested in the dynamics generated between the low lying doublon states and the $(0,0)$ lower energy band. We can induce Raman transitions between states in these bands using an external driving laser with Raman detuning $\Delta = 2E_1 - U - \omega$. The interaction between the (classical) radiation of amplitude Ω and frequency ω, and the system is $H_{\mathrm{rad}} = \Omega\cos(\omega t)\sum_i f_i^\dagger(c_i + \alpha(c_{i-1} + c_{i+1})) + \mathrm{h.c}$ with $\alpha = \frac{A_1}{A_0} \ll 1$. The amplitudes A_m decay fast with m as they represent the matrix element of the different Wannier functions and the laser radiation profile.

We couple our system to a 3-dimensional (3D) Bose Einstein Condensate (BEC). This coupling is realized by immersing the system in the BEC which acts as a dissipative, memoryless bath (valid when the spectral function of

the bath is almost constant around the frequency of the laser ω). The bath is very efficient to de-excite the system and does not induce excitations to higher energy bands as its temperature T satisfy $T \ll \omega$ so thermal excitations in the bath cannot induce excitations in the system. This type of bath has been used to obtain superconducting states realizing Majorana zero modes [1]. The quasiparticle excitations of this bath are described by the Hamiltonian $H_{\text{bath}} = \sum_k E_k b_{\mathbf{k}}^\dagger b_{\mathbf{k}}$, where the bogoliubov quasiparticles $b_{\mathbf{k}}$ have mass m_b and propagate with velocity c_b. These bosonic quasiparticles have energy $E_k = k(c_b^2 + k^2/4m_b^2)^{1/2}$,with $k = \sqrt{\mathbf{k}^2} = (k_x^2 + k_y^2 + k_z^2)^{1/2}$ the magnitude of the three dimensional momentum of the Bogoliubov quasiparticles. Here $b_{\mathbf{k}}$ ($b_{\mathbf{k}}^\dagger$) destroys (creates) a bosonic bogoliubov excitation of momentum $\mathbf{k}$. We work in the regime where the excitation induced by the laser is relaxed by the bath immediately, such that states with multiple excitations are not created. This approximation corresponds to the limit of weakly far detuned laser $E_d \gg |\Delta| \gg |g|, |\Omega|$.

The interaction between the system and the condensate is described by the density interaction $H_{\text{bath/sys}} = g \int d\mathbf{r} \delta\rho_s(\mathbf{r}) \delta\rho_{\text{bath}}(\mathbf{r})$ [2], where g is the strength of the system/bath coupling, which is assumed to be small. The density $\delta\rho_s(\mathbf{r})$ is the density of fermions at the point $\mathbf{r}$ in 3D, so it involves the Wannier wavefunctions of the fermions. The density $\delta\rho_{\text{bath}}$ corresponds to the phonon waves around the equilibrium BEC density induced by the interaction with the fermions in the optical lattice.[2] We move to a rotating frame of reference (the rotating wave approximation, RWA), where the problem, in the reduced Hilbert space which consists of the two lowest bands and the bath, looks static. This is achieved by introducing the time dependent unitary transformation $\mathcal{U}_t = \exp(iH_{\text{sys}}t)$. The penalty is that terms involving higher bands of the extended Hilbert space are fast oscillating. By coarse graining in time we are allowed to get rid of these terms, which amounts to projecting out the higher bands. The radiation Hamiltonian is modified by the rotating wave approximation accordingly. In this rotating basis, the interaction Hamiltonian with the bath $H_{\text{bath/sys}}$ is also modified as $H'_{\text{int}} = \mathcal{U}_t H_{\text{int}} \mathcal{U}_t^\dagger$. Retaining only the slow oscillating terms (with frequency $\sim \omega$) in the system-bath interaction, amounts to consider just the transition between the doublon and the $(0,0)$ band. Each of these transitions is accompanied by the creation (or destruction) of a phonon excitation in the bath.

Tracing over the bath, using the Born-Markov approximation, results in a quantum master equation for the effective density matrix in the reduced Hilbert space involving the bottom and the doublon bands. For small amplitude of the external drive Ω, such that $|\Delta| \gg |g|, |\Omega|$, no more than one doublon is excited at any given time. We may then trace out the doublon band, and obtain a closed dissipative equation of motion within the Hilbert space of the lowest band. After this adiabatic elimination of the doublon band, we find the quantum master equation $\dot{\rho} = \mathcal{L}(\rho) = \sum_{i=1}^{N_\Phi} \gamma \left(\tilde{\ell}_i \rho \tilde{\ell}_i^\dagger - \frac{1}{2}\{\tilde{\ell}_i^\dagger \tilde{\ell}_i, \rho\} \right)$, with $\gamma = \frac{\Omega^2 g^2}{\Delta^2}\Gamma_0$ and Γ_0 parameterizing details of the bath/system coupling. The quantum jump operator is in turn $\tilde{\ell}_i = R_i^\dagger \mathcal{Q}_i$ with $R_i^\dagger = c_i^\dagger c_{i+1}^\dagger + t(c_i^\dagger c_{i+2}^\dagger + c_{i+1}^\dagger c_{i+3}^\dagger)$, and $\mathcal{Q}_i = \ell_{1,i} + A(\ell_{0,i-1} + \ell_{0,i+1}) + B(\ell_{1,i+1} + \ell_{1,i-1})$.

[1] S. Diehl, E. Rico, M. A. Baranov, and P. Zoller, Nat Phys **7**, 971 (2011).
[2] S. Diehl, A. Micheli, A. Kantian, B. Kraus, H. P. Buchler, and P. Zoller, Nat. Phys. **4**, 878 (2008).
